# In Vivo Evidence That TRAF4 Is Required for Central Nervous System Myelin Homeostasis

**DOI:** 10.1371/journal.pone.0030917

**Published:** 2012-02-17

**Authors:** Sébastien Blaise, Marie Kneib, Adrien Rousseau, Frederic Gambino, Marie-Pierre Chenard, Nadia Messadeq, Martine Muckenstrum, Fabien Alpy, Catherine Tomasetto, Yann Humeau, Marie-Christine Rio

**Affiliations:** 1 Functional Genomics and Cancer Department, Institut de Génétique et de Biologie Moléculaire et Cellulaire, Centre National de la Recherche Scientifique UMR7104, Institut National de la Santé et de la Recherche Médicale U964, Université de Strasbourg, Illkirch, France; 2 Centre National de la Recherche Scientifique UPR3212, Strasbourg, France; 3 Département de Pathologie, Centre Hospitalier Universitaire de Hautepierre, Strasbourg, France; Nagasaki University Graduate School of Biomedical Sciences, Japan

## Abstract

Tumor Necrosis Factor Receptor-Associated Factors (TRAFs) are major signal transducers for the TNF and interleukin-1/Toll-like receptor superfamilies. However, TRAF4 does not fit the paradigm of TRAF function in immune and inflammatory responses. Its physiological and molecular functions remain poorly understood. Behavorial analyses show that TRAF4-deficient mice (TRAF4-KO) exhibit altered locomotion coordination typical of ataxia. TRAF4-KO central nervous system (CNS) ultrastructure shows strong myelin perturbation including disorganized layers and disturbances in paranode organization. TRAF4 was previously reported to be expressed by CNS neurons. Using primary cell culture, we now show that TRAF4 is also expressed by oligodendrocytes, at all stages of their differentiation. Moreover, histology and electron microscopy show degeneration of a high number of Purkinje cells in TRAF4-KO mice, that was confirmed by increased expression of the Bax pro-apoptotic marker (immunofluorescence), TUNEL analysis, and caspase-3 activation and PARP1 cleavage (western blotting). Consistent with this phenotype, MAG and NogoA, two myelin-induced neurite outgrowth inhibitors, and their neuron partners, NgR and p75NTR were overexpressed (Q-RT-PCR and western blotting). The strong increased phosphorylation of Rock2, a RhoA downstream target, indicated that the NgR/p75NTR/RhoA signaling pathway, known to induce actin cytoskeleton rearrangement that favors axon regeneration inhibition and neuron apoptosis, is activated in the absence of TRAF4 (western blotting). Altogether, these results provide conclusive evidence for the pivotal contribution of TRAF4 to myelination and to cerebellar homeostasis, and link the loss of TRAF4 function to demyelinating or neurodegenerative diseases.

## Introduction

Tumor Necrosis Factor (TNF) Receptor-Associated Factors (TRAFs), originally described by Goeddel and colleagues [1], constitute a family of adapter proteins containing 7 members that has been found in mammals, as well as in other multicellular organisms such as Drosophila [2], Caenorhabditis elegans [3], and zebra fish [4]. Mammalian TRAFs have emerged as the major signal transducers for the TNF receptor, Toll-like receptor and interleukin receptor superfamilies. A wide range of biological functions, such as adaptive and innate immunity, embryonic development, stress response and bone metabolism, are mediated by TRAFs via the induction of cell survival, proliferation, differentiation and death (reviewed in [5] [6]).

We first identified TRAF4 in human breast tumors [7]. TRAF4 orthologues have been reported in the mouse, rat, fly, fish and worm, suggesting that TRAF4 exerts important and conserved function(s). Although TRAF4 shares common protein features with other TRAFs, it does not behave like them. It is not involved in biological processes related to the immune system, as the other TRAF. We previously generated TRAF4-null mice (TRAF4-KO), and showed that TRAF4 deficiency in the mouse was lethal at embryonic stage in approximately one third of the homozygote mutants, mainly due to defects in neural tube closure [8]. Surviving animals manifest various alterations. All TRAF4-KO mice have a defect in the upper respiratory tract, with a striking reduction of the diameter of the tracheal lumen, leading to a wheezing sound [8], [9]. Three to six of the upper tracheal rings below the cricoid cartilage are frontally interrupted and sometimes fused. The disorganization of the upper respiratory tract extends to the level of the stem bronchi below the tracheal bifurcation [8]. Other non-fully penetrant phenotypes including various malformations of the axial skeleton (ribs, sternum, tail), and mild spina bifida were also observed [8]. Recently, TRAF4 was confirmed to be an essential gene for neural crest development and neural folding in Xenopus [10]. Unlike other TRAF-deficient mice, TRAF4-KO mice exhibit normal immune response [11].

Our current knowledge of the physiological role of TRAF4 remains poor and its molecular function is largely unknown [12] [13] (reviewed in [14] [15]) [16]. TRAF4 has notably been shown to be involved in the subcellular localization of reactive oxygen species (ROS) products in endothelial cells via its binding to the p47phox protein [17], in the maintenance of epithelial cell polarity via a function at the tight junction (TJ) level [18], and in the migration of dendritic cells [11]. Moreover, TRAF4 has been shown to be overexpressed in numerous human carcinomas and an oncogenic role has been proposed for TRAF4 [7], [19]. Interestingly, TRAF4 expression was observed in several regions of the CNS. Thus, during embryogenesis, a high level of expression is observed during the ontogenesis of the mouse [20] and Zebrafish [4] CNS. In drosophila, DTRAF1 (fly TRAF4 orthologue) accumulates in mesodermal cells and neural precursors and is correlated with the onset of morphogenetic and cellular movements [21]. Strong TRAF4 expression is also observed in several regions of adult mouse CNS. In all cases, it has been reported to be expressed by neurons, and notably in the Purkinje cells of the cerebellum [20]. Collectively, all these data suggest that TRAF4 might exert a function related to the nervous system.

Here we show that TRAF4-KO mice exhibit altered coordination of locomotion, typical of ataxia. We find that, in addition to neurons, TRAF4 is also expressed in oligodendrocytes from early progenitors to mature myelinating cells. We demonstrate a dramatic alteration of the myelin ultrastructure and a degeneration of a high number of Purkinje cells in the absence of TRAF4. Molecular analyses indicate the activation of the NgR/p75NTR/RhoA signaling pathway, triggered by overexpression of the MAG and NogoA myelin-induced neurite outgrowth inhibitors. Our findings show that TRAF4 is required for myelin integrity and Purkinje cell survival, and link the loss of TRAF4 function to brain diseases.

## Materials and Methods

### Ethics statement

All mice were housed in an animal facility licensed by the French Ministry of Agriculture (agreement no. B67-218-5), and all animal experiments were supervised by MC. Rio (agreement no. 67-194 to MC. Rio, approved by the Direction des Services Vétérinaires, Strasbourg, France), in compliance with the European legislation on care and use of laboratory animals.

### Animals and sample collection

Homozygous TRAF4-KO [8] or WT 129SvPas littermate pups were maintained under standard laboratory conditions. Mice were sacrificed by excess halotane except for electrophysiology analysis where animals were decapitated under isoflurane general anaesthesia. Tissues were immediately incubated in 4% paraformaldehyde (PFA) or frozen in liquid nitrogen.

### Oligodendrocyte culture

Primary culture of mouse oligodendrocytes was carried out by differential adhesion as described previously [22]. Briefly, 3–4 day postnatal mouse cerebella were dissected and cleared of meningeas. After mechanical dissociation in Dulbecco's modified Eagle's medium (DMEM) through a nylon sieve (82 micron), the suspension was maintained in DMEM supplemented with sodium bicarbonate (25 mM), insulin (0.5 µM), gentamycin (0.05 mg/ml), and 10% calf serum. Cells were plated in poly-D-lysine-coated culture flasks and incubated at 37°C in a water-saturated incubator equilibrated with 5% CO2. Medium was changed every 3 days. After 10 days, oligodendrocyte progenitor cells (OPC) fixed on an astrocyte layer were obtained. OPC were detached mechanically by gentle buffer flow. The obtained OPC were then plated in 10 cm diameter plates (without poly-D-lysine coating) and incubated for 2 h at 37°C to eliminate contaminating cells. This step was repeated 3 times. Finally, OPC were plated in poly-D-lysine-coated culture flasks at 0.3 to 1×10^6^ cells/ plate (35 mm diameter). After a few hours, the cells were rinsed and exposed to a serum-free, chemically defined medium composed of DMEM supplemented with bovine transferrin (10 µg/ml), progesterone (20 nM), bovine insulin (5 µg/ml), putrescine (100 µM), sodium selenite (30 nM), penicillin (50 UI/ml), D-glucose (5 g/l), T3 (0.1 µM) and streptomycin (50 µg/ml) to allow cell differentiation. The medium was completely renewed every 1 day. Cells were allowed to differentiate for 1, 2 and 4 days. OPC and differentiating cells were directly harvested for RNA extraction.

### Western blotting

Proteins were extracted from tissues (4°C, RIPA buffer, Proteases inhibitor cocktail) (Sigma Chemicals, St Louis, MO). Twenty µg of proteins were analyzed on 10% SDS–polyacrylamide gel. After electrotransfer in Tris–glycine buffer (48 mM Tris base, 39 mM glycine, 0.037% SDS and 20% ethanol; 100 mV, 60 min), membranes (PVDF, New England Nuclear, Boston, MA) were blocked (5% bovine serum albumin, Tris-buffered saline (TBS), 1 h). Blots were then probed (overnight, 4°C) with appropriate primary antibodies (listed in Table 1). After washing (TBS, 0.1% Tween 20), peroxidase-conjugated secondary antibody (40 mU/ml, Boehringer), luminol chemiluminescent substrate (Boehringer, Mannheim, Germany) and Kodak X-Omat films (Perkin-Elmer, Courtaboeuf, France) were used.

**Table 1:** Antibodies used for immunofluorescence and western blotting.

Antibody	Immuno-fluorescence	Western blotting	Source	Suppliers
Bax	1/500	-	Rabbit	Santa Cruz Biotechnology
Calbindin D 28K	1/1000	-	Mouse	Swant
Caspase-3 uncleaved	-	1/1000	Rabbit	Chemicon
Caspase-3 cleaved	-	1/1000	Rabbit	Sigma
Claudin-11	-	1/500	Rabbit	USBiological
MAG	-	1/1000	Rabbit	Santa Cruz Biotechnology
MBP	-	1/500	Mouse	USBiological
NogoA (H300)	-	1/1000	Rabbit	Santa Cruz Biotechnology
P75NTR	-	1/1000	Rabbit	Upstate
PARP	-	1/1000	Rabbit	Santa Cruz Biotechnology
Rock 2	-	1/1000	Goat	Santa Cruz Biotechnology
P-Rock 2	-	1/500	Rabbit	Rockland
TRAF4	-	1/1000	Rabbit	IGBMC
Tubulin	-	1/2000	Mouse	Sigma

### Histopathological and immunofluorescence analyses

Four-micrometer sections of paraffin-embedded samples were stained with hematoxylin-eosin (HE), cresyl violet or Luxol fast blue. Cells were counted in six section areas delineated by an ocular grid of 1/400 mm2 (×40 magnification). Tissue sections were counterstained by the 4,6-diamidino-2-phenylindole fluorescent dye (Dapi, 0.5 µg/mL in PBS, Sigma). For immunofluorescence, tissue was first denatured by incubating CNS sections in 2 N HCl for 45 min at room temperature followed by 10 min neutralization in 0.1 M sodium borate at pH 8.5. Tissue was rinsed in PBS for 10 min and then in PBS containing 10% bovine serum for 1 h and incubated overnight at 4°C with primary antibodies (Table 1). Tissue was incubated for 1 h with the secondary antibody (IgG conjugated to AlexaFluor, 1/1000; Molecular Probes, Cergy Pontoise, France) in PBS containing 10% bovine serum [23]. Immunofluorescence was analysed by confocal microscopy. TUNEL staining was performed using the Apoptag Apoptosis Detection Kit (Millipore Corp). Sections were counterstained with nuclear fast red.

#### Electron microscopy

The tissue samples were fixed (2.5% glutaraldehyde in cacodylate buffer, overnight, 4°C), washed for 30 min in the same buffer and then post-fixed (1 h, 1% buffered osmium tetroxide) [24]. Samples were dehydrated with gradient concentrations of ethanol and embedded in Epon 812. Ultrathin sections were cut, stained with uranyl acetate and lead citrate before analysis (Morgagni 268D microscope). Our experiments included 6 WT and 6 TRAF4-KO mice. For each of them and for each tissue, 3 grids were done and at least 6 areas were observed for each grid (a total of more than 100 images).

### RNA preparation and quantitative RT-PCR

RNA was extracted using Rneasy® Lipid Tissue (Qiagen S.A., France) and converted to cDNA with SuperScript™ II reverse transcriptase (Invitrogen™ lifetechnologies) and oligo dT24 primers. Quantitative RT-PCR (Q-RT-PCR) was performed using SYBR® Green JumpStart PCR kit (Sigma). 36B4, 18S, GAPDH mRNA were used as internal controls. RNA tested and primer sequences are listed on Table 2.

**Table 2:** Primers used for Q-RT-PCR.

mRNA	Sense probe (5′-3′)	Antisense probe (5′-3′)
18S	GGGAGCCTGAGAAACGGC	GGGTCGGGAGTGGGTAATTTT
36B4	AGATTCGGGATATGCTGTTGG	AAAGCCTGGAAGAAGGAGGTC
Caspr	CGCCATGACCTTCACTACCACTT	GCCATATCGATCCACCCGCACAT
CNPase	AGACAGCGTGGCGACTAGACT	GGGCTTCAGCTTCTTCAGGT
GAPDH	ACCACAGTCCATGCCATCAC	TCCACCACCCTGTTGCTGTA
MAG	ACCATCTCAGCCTTCGAGGGCA	TGTCCAGGACGCTGTGCTCCGA
MBP	CCTGCCCCAGAAGTCGC	CTTGGGATGGAGGTGGTGTTC
NF68	CCCCTCTGAAGGAGAAGCA	TCTTTTGTGTCTTCAGACTCATCC
NgR	AGAGGTTGTTGGCAAACAGGTAG	ATCTTCCTGCATGGCAACCGAAT
NogoA	CAGGTGATGTACGCTCTGGA	TGAGGGAAGTAGGGATGTGC
Olig2	GCGGTGGCTTCAAGTCATCTT	CGGGCTCAGTCATCTGCTTCT
OmgP	ACCTCAAGCTTATTTACTATGAAG	AGTGTTTCCATTTGCAGTGGTT
PDGF-Ra	GACGTTCAAGACCAGCGAGTT	CAGTCTGGCGTGCGTCC
PLP	GCAAGGTTTGTGGCTCCAAC	CGCAGCAATAAACAGGTGGAA
P75NTR	GCGCCACCGAGCCGTCAAGC	CGTCAGAGCCCTCCGGGGGCG
TRAF4	GCCTTTGTTCTCTGCCCTTT	TCCCATTGCCAGCTTAGG

### Behavioral analyses

Mice were weighed before use. For all tests, mice were given 3 trials separated by 10 min intertrial intervals. The mean of these consecutive trials was taken.

#### Negative geotaxis

[23]: reflexes were tested as previously described. Mice were positioned with the head facing down on an inclined plane with a 20% slope. The time needed for mice to turn completely and reach a position with the head facing up was measured. The duration of the test was limited to 120 sec.

#### Wooden Beam

[25]: mice were placed in the center of a rectangular wooden beam (length, 110 cm; width, 5.5 cm; thickness, 3.5 cm) divided into 11 segments and situated at 75 cm from floor. The number of crossed segments and fall latency (cut-off 60 sec) were recorded.

#### Wooden Edge

[25]: mice were placed at edge center (length, 90 cm; width, 1 cm), divided into 9 segments, at 75 cm from floor. The number of crossed segments and fall latency (cut-off 60 s) were recorded.

#### Rotarod

[26]: motor performance was evaluated using a rotarod apparatus (Bioseb, Chaville, France). Mice were placed on the rod (3 cm diameter) under constant acceleration (4 to 40 rpm at a rate of 0.1 rpm/sec). Fall latency was measured.

#### Locomotion/equilibrium test

[23]: this test comprises 3 phases: swimming in a round container (15 cm diameter, 23 cm height), climbing along a vertical metal rod (6 mm diameter, 20 cm) whose base was inside the water, and reaching a platform. The time necessary for the mice to reach the platform with all four paws after being put inside the water was recorded (cut-off 300 sec). The percentage of mice that succeeded in each group were given.

#### Grip test

[27]: the animal with its forelimbs holding onto a grid (Bioseb, Chaville, France) was slowly moved backwards until the grip was released. The dynamometer records the maximal strength developed.

#### Suspension test

[23]: mice were suspended by the forelimbs on a metal rod (1 mm diameter, 20 cm above the table). Fall latency was recorded.

#### Open field

[27]: mice were analyzed for 30 min in a square arena (44×44×17 cm). Motor activity (distance traveled and the time spent in movement) were automatically recorded (Panlab, Actitrack, Barcelona, Spain).

#### Touch test

[28]: mice were placed under plastic chambers (3×8×12 cm) on top of a wire mesh-top table and allowed to acclimate for 5 min. The number of withdrawals in response to non-noxious mechanical stimuli using calibrated von Frey monofilaments of increasing force (0.008 to 4 g) (Bioseb, Chaville, France) were recorded (six applications).

#### Hot plate test

[29]: mice were placed on a 52°C hot plate and the latency of forelimb licking were measured.

### Electrophysiology

Standard procedures were used to prepare 300 µm thick parasagittal slices from 8-day-old and 90-day-old (P8–P90) mice [30]. Briefly, brains were dissected in ice-cold artificial cerebrospinal fluid (ACSF), and sliced with a vibratome at 4°C. Slices were maintained for 30 min at 32°C in an interface chamber containing ACSF equilibrated with 95% O2/5% CO2 and containing (in mM): 124 NaCl, 2.7 KCl, 2 CaCl2, 1.3 MgCl2, 26 NaHCO3, 0.4 NaH2PO4, 10 glucose, 4 ascorbate, and then for at least 1 hour at room temperature before being transferred to a superfusing recording chamber. Whole-cell recordings from Purkinje neurons were performed at 33°C, in a chamber perfused at 2 ml/min with bubbled ACSF. Patch electrodes (3–5 MΩ) were pulled from borosilicate glass tubing and filled with a solution containing (in mM): 140 Cs-methylsulfonate, 5 QX314-Cl, 10 HEPES, 10 phosphocreatine, 4 Mg-ATP, and 0.3 Na-GTP (pH adjusted to 7.25 with CsOH, 295 mOsm). Monopolar stimulation electrodes were pulled from borosilicate glass tubing and filled with ACSF. Stimulation pulses (1 ms, 1 to 30 µA) were applied using an externally triggered stimulus isolator device (World Precision Instruments). Data were recorded with a Multiclamp700B (Axon Instruments), filtered at 2 kHz and digitized at 10 kHz. In all experiments, series resistance was monitored throughout the experiment by applying a hyperpolarizing current, and if it changed by more than 15% or overwhelmed 25 MΩ, the data were not included in the analysis. Data were acquired and analyzed with: ClampEx10.2, ClampFit10.0 (Axon Instruments), SigmaPlot 11 software and R language and environment for statistical computing. Spontaneous Inhibitory PSCs (sIPSC) were automatically detected by a template-based routine in the ClampFit 10.0 software. Evoked IPSC and EPSC amplitude were determined as the maximal current obtained following stimulation within the molecular layer by holding the Purkinje cell at +10 mV and −70 mV respectively. All values are given as means ± standard error of the mean (SEM) and statistical procedures performed using R language and environment for statistical computing. Mean values were compared between genotypes using either unpaired Student's t test or Mann-Whitney test as appropriate. QX314 was purchased from Alomone labs.

### Statistical Analyses

Data were prospectively collected and analyzed with Statview 5 software for Windows (SAS Institute, Berkley, CA). Reported as means±SD, raw data were compared by using one-way analysis of variance with Fisher's test. Regarding behavioral studies, the distribution of the data deviated quite strongly from normality and variances were not equal. Therefore, nonparametric statistics were used and data were analyzed by Student's t test and Mann-Whitney U-test. For all analyses, at least three independent experiments (each of them including at least 6 mice) were performed. p values lower than 0.05 were considered as significant (*, p<0.05; **, p<0.01; ***, p<0.001).

## Results

### TRAF4 deficiency alters locomotion coordination

A panel of standard neurobehavioral tests for motor and coordination performance [31] [23] was performed on TRAF4-KO and wild-type (WT) littermate mice. There is no difference between WT and TRAF4-KO mouse body weights whatever their age (Table 3). All results are summarized in Table 4. The geotaxis test was first performed on 8- (closed eyes) and 21- (opened eyes) day-old mice. At both ages, the time needed to turn up completely was significantly increased in TRAF4-KO compared with WT mice (day 8: p<0.001 and day 21: p = 0.002). This is also true for 6-month-old mice (p<0.011). These data suggest that TRAF4-KO mice exhibit alterations in anterior limb coordination and/or brain maturation. The Rotarod test performed on adult mice (8 weeks and 6 months) showed that TRAF4-KO mice stayed a shorter time on the rod compared with WT (p = 0.011 and p = 0.010). Moreover, the rod speed at fall moment was slower in TRAF4-KO mice (p = 0.007 and p = 0.029), confirming alterations in movement coordination in adult mice in the absence of TRAF4. We therefore investigated the equilibrium capacity using the wooden edge test. TRAF4-KO mice stayed less time at the edge (p = 0.004) and crossed lower number of segments (p<0.001) compared with WT. Moreover, using the wooden beam test, TRAF4-KO mice crossed less segments than control mice (p = 0.016), although the fall latency was similar (p = 0.273). These data show that TRAF4-KO mice exhibit an equilibrium deficiency. A locomotion coordination and equilibrium test including swimming, climbing and landing gave similar results. At 8 weeks, four-fold more WT mice successfully achieved the task compared with TRAF4-KO mice (p = 0.004), a difference which was not due to decreased muscular force in the absence of TRAF4 since the grip and suspension tests gave similar results in TRAF4-KO and WT mice (p = 0.060 and p = 0.111, respectively). Similar results were obtained at 6 months. Thus, TRAF4 is important for the correct coordination of limbs during exercise, suggesting that impaired TRAF4-KO locomotion/equilibrium might result from an alteration in the cerebellum. Indeed, several studies have shown that the adult cerebellum is implicated in equilibrium and posture [25]. This behavioral phenotype is not greatly amplified with age (6 months). Since the cerebellum is involved in agonist and antagonist muscle coordination and reflex control [32]–[33], we directly explored these functions by performing the touch test and the hot-plate test. The touch test showed that TRAF4-KO mice were less sensitive to pressure from von Frey monofilaments than WT mice (p = 0.019). Moreover, the latencies for forepaw licking in the hot plate test were significantly increased (p = 0.038). Finally, we studied the exploration capacity of TRAF4-KO mice using the open field test. The number of movements recorded, the time and the total distance covered by TRAF4-KO mice were reduced compared with control mice (p = 0.044, p = 0.008 and p = 0.013, respectively). Collectively, all these symptoms indicate that TRAF4 deficiency induces an alteration in locomotion coordination typical of ataxia.

**Table 3:** Body weight of WT and TRAF4-KO mice at various ages.

Age/nb	WT (g)	TRAF4-KO (g)	p-value
8 d (n = 18)	15.8±0.4	16.5±0.5	0.3276
21 d (n = 18)	18.2±0.2	18.5±0.1	0.2660
8 w (n = 18)	21.9±0.9	22.1±0.2	0.8690
6 m (n = 8)	29.2±1.2	26.7±2.0	0.2873

Data are means±SD; g, gram; n, number of mice/group; d, day; w, week; m, month.

**Table 4:** Results of locomotion and behavior tests in WT and TRAF4-KO mice.

Test	Age/nb	Parameters	WT(n = 18)	TRAF4-KO	p-value
Geotaxis	8 d (n = 8)	Time (s)	7.1 (3.1)	20.2 (18.6)	0.0007
	21 d (n = 8)	Time (s)	6.8 (6.0)	18.3 (40.5)	0.0019
	6 m (n = 8)	Time (s)	8.5 (4.5)	13.7 (15.0)	0.0112
Rotarod	8 w (n = 18)	Fall latency (s)	149.5 (65.0)	89.0 (31.5)	0.0111
		Speed (rpm/min)	13.0 (2.0)	9.0 (1.5)	0.0068
	6 m (n = 8)	Fall latency (s)	92.9 (17.6)	76.0 (20.7)	0.0104
		Speed (rpm/min)	10.0 (2.5)	7.0 (1.5)	0.0286
Wooden edge	8 w (n = 18)	Fall latency (s)	389.5 (441.8)	28.7 (134.8)	0.0045
		Segment (nb)	5.0 (0.0)	3.0 (1.5)	0.0005
Wooden beam	8 w (n = 18)	Fall latency (s)	720.0 (0)	720.0 (0.0)	0.2733
		Segment (nb)	11.0 (21.0)	6.0 (0.0)	0.0158
Locomotion/equilibrium	8 w (n = 18)	Success (%)	61.5	15	0.0036
	6 m (n = 8)	Success (%)	37.5	0	<0.001
Grip	8 w (n = 18)	Force (N)	95.0 (37.0)	72.1 (38.0)	0.0604
	6 m (n = 8)	Force (N)	115.0 (49.0)	104.5 (19.0)	0.2264
Suspension	8 w (n = 18)	Time (s)	96.3 (12.5)	76.9 (8.8)	0.1110
	6 m (n = 8)	Time (s)	35.6 (43)	12.2 (22.0)	0.0033
Touch	8 w (n = 18)	Pressure (g)	0.5 (0.8)	1.2 (2.5)	0.0193
Hot-plate	8 w (n = 18)	Latency (s)	36.1 (7.8)	46.5 (13.3)	0.0378
Open field	8 w (n = 18)	Movement (nb)	313.0 (450.5)	105.0 (315.5)	0.0440
		Time (s)	183.0 (280.1)	1.3 (34.9)	0.0078
		Distance (cm)	1632.1 (3089.7)	60.2 (35.2)	0.0135

Tests were performed with animals aged 8-day-old, 21-day-old, 8-week-old and 6-month-old as indicated. Values correspond to medians with interquartiles in parentheses. Statistical analysis was two-tailed Mann-Whitney U-test. S, second; n, number of mice of each set (WT and TRAF4-KO) involved in the test; nb, number; %, percentage; N, Newton; cm, centimeter.

### TRAF4 deficiency induces myelin alteration

**Figure 1:**
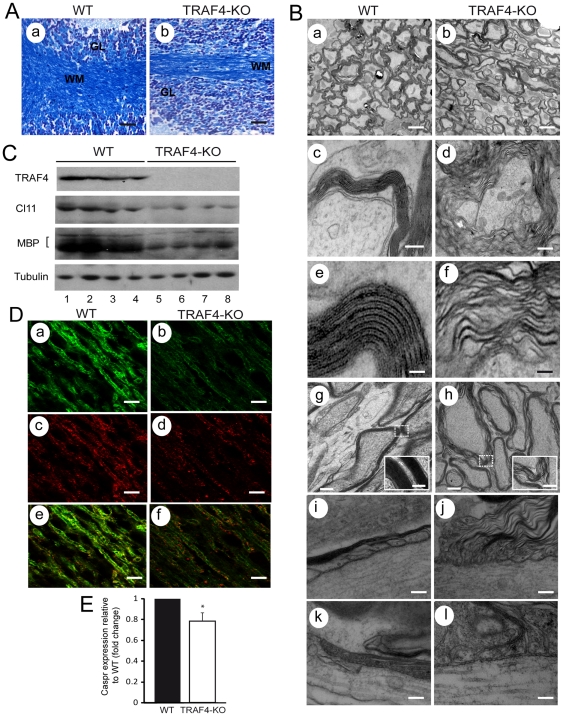
Myelin alteration in 8-week-old TRAF4-KO mice. (A): Histological analysis of myelin using Luxol fast blue staining of the cerebellum from WT (a) and TRAF4-KO (b) mice showed a lower amount of myelin in the absence of TRAF4 (n = 6/group). (B): (a–h) Electron microscopy analysis on transversal section of WT (a, c, and e) and TRAF4-KO (b, d and f) cerebellum. The continual wrapping of the oligodendrocyte cell myelin membrane forms a multilamellar layer around axons that is electron dense. The number of myelinated axons was lower and they were not in close contact with each other in the absence of TRAF4 (b versus a). Note the loss of organization of myelin sheath in TRAF4-KO cerebellum (b, d and f) compared with WT mice (a, c and e) suggesting defects of myelin TJs. Similar myelin abnormalities were observed in the TRAF4-KO optic nerves (h) compared with WT mice (g). (i–l) : Electron microscopy analysis on longitudinal section of WT (i, k) and TRAF4-KO (j, l) paranodes. WT paranodal myelin was well organized, and electron-dense TBs between the axonal and oligodendrocyte membranes were present in the cervical region of the spinal cord (i) and the optic nerve (k). In contrast, in the TRAF4-KO spinal cord (j) and optic nerve (l), some paranodal loops showed disorganization. Myelin membranes were separated from the axon and from each other. Paranodal loops oriented away from the axon were also observed. TBs are sometimes disordered or absent. Bars: a and b, 1 µm; c and d, 0.5 µm; e and f, 0.2 µm; g and h: 1 µm, inset 0.5 µm; i–l: 0.25 µm. (C): Western blot analysis of the myelin-specific MBP marker and of the interglial-specific TJ marker claudin 11 (cl 11) showed a decreased expression level of both proteins in 4 independent TRAF4-KO cerebellum protein extracts (lanes 5–8) compared with 4 WT samples (lanes 1–4) ; tubulin served as a loading control. (D): MBP (a and b) and claudin 11 (c and d) immunofluorescence of adjacent sections of WT and TRAF4-KO cerebellum, respectively, showed a lower expression of both protein in the absence of TRAF4. (e and f): merge of the MBP-stained and claudin 11-stained adjacent sections. Bars: a–f, 10 µm. (E): Q-RT-PCR analysis of expression of the specific marker of axoglia TJs caspr using total RNA extracts from WT (black) and TRAF4-KO (white) cerebellum showed a decrease of caspr expression in the absence of TRAF4. Data are means±SD (n = 6/group) (ANOVA, Fisher's test).

Myelin plays a key role in the functioning of the nervous system by allowing fast saltatory conduction of nerve pulses. Since it has been reported that myelin disorganization leads to alterations in locomotion coordination and/or severe ataxia [34]–[35], CNS myelin organization was examined in WT and TRAF4-KO mice. Histological analysis by Luxol fast blue staining, a marker of myelin, showed that the myelin network in the cerebellum white matter was disorganized and less dense in TRAF4-KO mice compared with WT (Figure 1A). Electron microscopy was performed on 6 WT and 6 TRAF4-KO 8-week-old mice, investigating both internodal and paranodal regions. We observed major ultrastructural alterations in the myelin of TRAF4-KO mice (Figure 1B). The number of myelinated axons was reduced when compared with WT mice (84.4±6.6 *vs* 97.9±5.1%, p = 0.0005). Moreover, in the WT brain, the adjacent axons were in contact with each other, while spaces between the axons were observed in the TRAF4-KO brain (Figure 1B a and b). The myelin layers surrounding the axons showed a loss of parallelism in the TRAF4-KO brain compared with WT (Figure 1B c–f). We then analyzed the ultrastructure of the optic nerve, another CNS component. Similar myelin alterations were observed (Figure 1B g and h), indicating that the myelin abnormalities due to the TRAF4 absence were not restricted to the cerebellum. Finally, the node of Ranvier, where lamellae of compact myelin sheats normally terminate in glial loops to establish the paranodal axoglial septate-like junctions [36], were also altered. In the WT cervical spinal cord and optic nerve we found the regular, densely stained transverse bands (TBs) in almost all of the paranodes examined between the paranodal loop and axon. Moreover, the paranodal loops contact the axon (Figure 1B, i and k). In contrast, although the majority of the TRAF4-KO paranodes exhibited correct morphology, 10–20% showed dramatic defects (Figure 1B, j and l). Thus, the attachement of the paranodal loops to the axon, and the space between the loops and axon may be increased. We observed loops completely detached and even everted, facing away from the axonal membrane. Finally, spacing between TBs may be irregular, or paranodal loops or portions of loops may lack well-defined TBs. We could not find any similar paranodal myelin alterations in the WT mice. Similar myelin phenotype was observed in older animals TRAF4-KO mice (6-month-old).

We then checked for myelin molecular abnormalities in TRAF4-KO cerebellum. Consistent with our ultrastructural observations, using western blot and immunofluorescence analyses, we observed a reduction in myelin basic protein (MBP), the second most abundant protein in the CNS myelin [37] (Figures 1C and 1D b and f). Moreover, it has been reported that myelin organization around the axons is maintained by tight-junction-like structures (TJ) located first between the lamellae of myelin sheaths and second, between the axon and the inner myelin loop. The observed TRAF4-KO myelin ultrastructural phenotype suggests that these structures might be altered in the absence of TRAF4. We then checked for the presence of oligodendrocyte-specific protein/Claudin-11 (OSP/cl11) and contactin-associated protein (Caspr ; also known as paranodin) proteins that are known to be respectively involved in these types of TJs [34] [38]. The TRAF4-KO cerebellum exhibited a reduction in claudin 11 protein compared with WT (Figures 1C and 1D d and f). Moreover, Q-RT-PCR of total RNA extracts from the cerebellum showed that caspr expression was also reduced by 20% compared with WT (0.8±0.1 fold change; p = 0.0257) (Figure 1E). These results show that both axoglial and interglial TJs, necessary for the correct organization and stabilization of myelin around axons, are altered in the absence of TRAF4.

### TRAF4 is normally expressed in the oligodendrocyte

**Figure 2:**
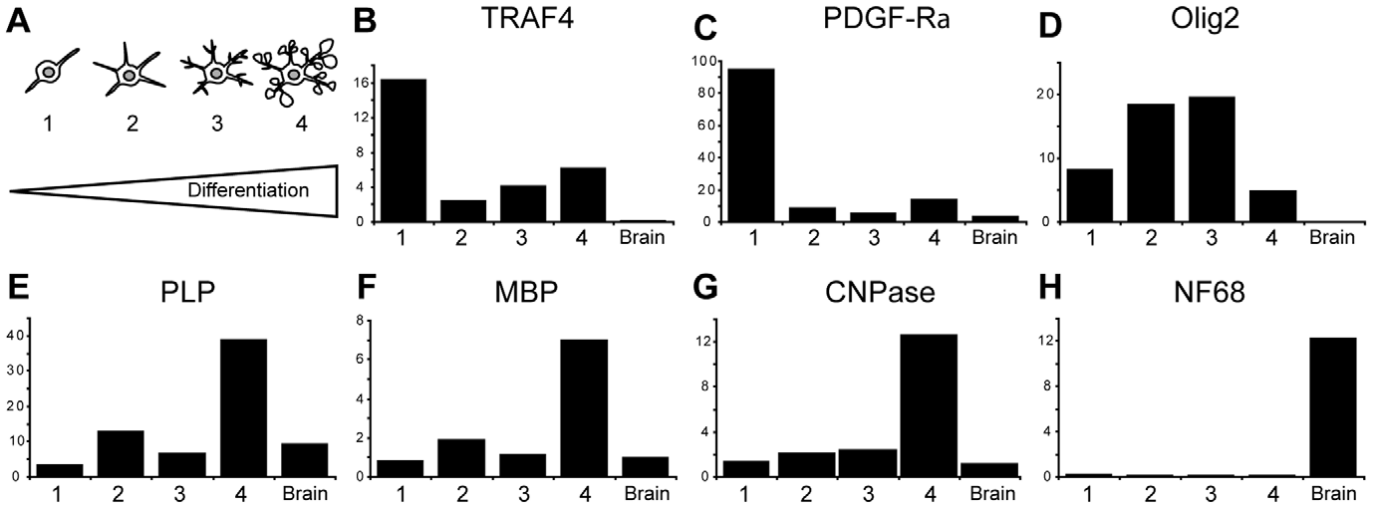
TRAF4 expression in WT oligodendrocyte primary cultures. (A): Oligodendrocyte primary culture and differentiation: schemes of the morphological characteristics of the 4 stages of differentiation obtained: early OPC, late OPC, pre-myelinating and myelinating oligodendrocytes (numbered from 1 to 4); (B–H): results of Q-RT-PCR analyses of RNA extracted from cells at each stages (relative expression compared with that of the GAPDH gene as control); (B) pattern of TRAF4 expression, (C–F) patterns of expression of the oligodendrocyte markers PDGF-Ra, Olig2, PLP, MBP and CNPase, respectively. NF68 expression pattern (H) in total adult brain extract (brain) serves as cell culture purity control. TRAF4 is expressed at all stages of oligodendrocyte differentiation.

Neurons and myelinating glia communicate with each other bidirectionnally in multiple ways to orchestrate axon myelination [39] [40]. The myelin phenotype of the TRAF4-KO animals suggests that TRAF4 might exert a key physiological function in the CNS myelination. We therefore studied the expression of TRAF4 in oligodendrocytes that are CNS cells producing myelin proteins. Oligodendrocyte progenitor cells (OPC) were extracted and purified from 3 to 4-day-old mouse brains. After in vitro differentiation, four stages (numbered 1–4, from the least to the most differentiated one) were obtained corresponding to early OPC, late OPC (preoligodendrocytes), immature (pre-myelinating), and mature (myelinating) oligodendrocytes, as characterized by their specific morphologies [41]. Thus, from a bipolar phenotype (stage 1), cells extend multipolar short processes (stage 2), long ramified branches (stage 3), and finally myelin membranes (stage 4) (Figure 2A). Total RNA was extracted from cells at each stage and analyzed by Q-RT-PCR for the expression of platelet-derived growth factor receptor alpha (PDGF-Ra), oligodendrocyte lineage-specific basic helix-loop-helix transcription factor (Olig2), 2′,3′-cyclic nucleotide 3′-phosphodiesterase (CNPase), ProteoLipid Protein (PLP) and (MBP) known markers of oligodendrocyte differentiation [41] (Figure 2). As previously described, high PDGF-Ra expression was observed at stage 1 (C), Olig2 was shown at all stages (D), whereas PLP, MBP and CNPase were mainly expressed at stage 4 (E–G). These patterns of expression confirmed the four morphologically-identified oligodendrocyte differentiation stages. Since TRAF4 is expressed by neurons, we also tested the expression of Neurofilament 68 (NF68), a marker of neurons [42]. The very low level of NF68 expression observed in primary cell cultures compared with that in the adult mouse brain extract (H) indicated that oligodendrocyte cultures were not contaminated by neurons. Thus, TRAF4 was expressed in all stages of differentiation with highest expression in early OPC (B). Together, these data show that TRAF4 is indeed expressed in oligodendrocytes.

### TRAF4 deficiency leads to Purkinje cell degeneration

**Figure 3:**
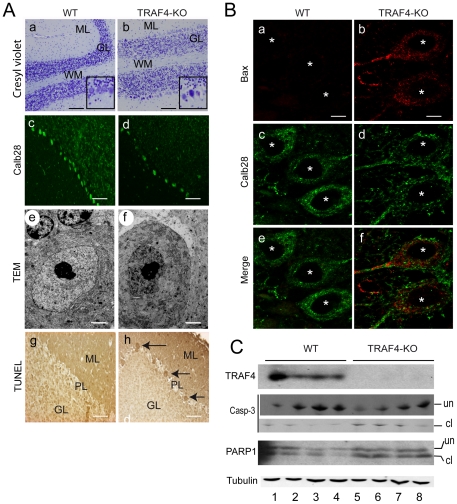
Purkinje cell degeneration in TRAF4-KO cerebellum. (A): analyses of WT (a, c, e and g) and TRAF4-KO (b, d, f and h) cerebellum at 15 postnatal days; (a, b) cresyl violet staining of parasagittal sections (ML, molecular layer; GL, granular cell layer; WM, white matter); (c, d) white matter immunofluorescence analysis of calb28 (green); In the absence of TRAF4, the number of Purkinje cells was decreased. (e, f) EM ultrastructural analysis: Purkinje cell nuclei exhibit classical morphological alterations specific of apoptosis notably chromatin condensation and nuclear membrane blebbing. (g, h) TUNEL staining: In the absence of TRAF4, the number of apoptotic Purkinje cells was increased (brown, arrows). Bars: a–d, g and h, 30 µm, insets 2-fold increase; e and f, 3.5 µm. (B): Bax immunofluorescence of WT and TRAF4-KO Purkinje cells showed a higher expression of this pro-apoptotic factor in the absence of TRAF4 (white stars, cell nuclei). Bars: a–f, 10 µm. (C): Western blot analysis of total cerebellum protein extracts from 4 WT (lanes 1–4) and 4 TRAF4-KO (lanes 5–8) 8-week-old mice. Both caspase 3 and PARP-1 cleaved forms were increased in the absence of TRAF4. cl, cleaved forms; un, uncleaved forms. Tubulin served as a loading control.

Since it is well documented that structural/functional abnormalities of the myelin sheath may cause axon degeneration and neuronal death [43], we hypothesize that the Purkinje cells might be altered in TRAF4-KO mice. The mouse cerebellum matures massively during the first month of life. Therefore, we first studied the 15-day-old mouse cerebellum (Figure 3A). Histological analysis using Cresyl violet staining showed several alterations, notably a less compact granular layer, a less cohesive Purkinje cell layer, and a Purkinje cell body with altered morphology (reduced cell size, condensed nuclei) in the absence of TRAF4 (a and b, and insets). Calbindin 28K (Calb28), a marker for Purkinje cells [44], immunostaining revealed obvious gaps in the Purkinje cell layer in the TRAF4-KO brain compared with WT (c and d), suggesting Purkinje cell degeneration in the absence of TRAF4. Accordingly, TRAF4-KO Purkinje cell nuclei presented altered ultrastructural features, namely condensed chromatin and blebbing of the nuclear membrane (e and f), as previously described in apoptotic cells [45]. Moreover, Bax immunofluorescence analysis showed an increased expression of this pro-apoptotic factor [46] in TRAF4-KO Purkinje cells (Figure 3B). Accordingly, we observed a high number of apoptotic Purkinje cells in the TRAF4-KO brain using TUNEL staining (Figure 3A g and h). Consistently, histological analysis of the adult cerebellum (8-week-old mice) showed that the number of Purkinje cells was lower in the TRAF4-KO brain compared with WT (101.7+/−29.1 versus 176.6+/−64.3; p<0.0001). Furthermore, the activation of caspase-3, an apoptosis effector, in TRAF4-KO mice was observed by western blot analysis of total cerebellum protein extracts (Figure 3C). Finally, cleavage of the caspase-3 nuclear target, Poly ADP-ribose polymerase-1 (PARP1), was also increased in the TRAF4-KO cerebellum compared with WT (lanes 5–8 versus lanes 1–4). These data show that TRAF4 deficiency induces apoptosis in the cerebellum, notably affecting Purkinje cells.

### The functional organization of neuronal activity in the remaining Purkinje cells of TRAF4-KO mice is not altered

**Figure 4:**
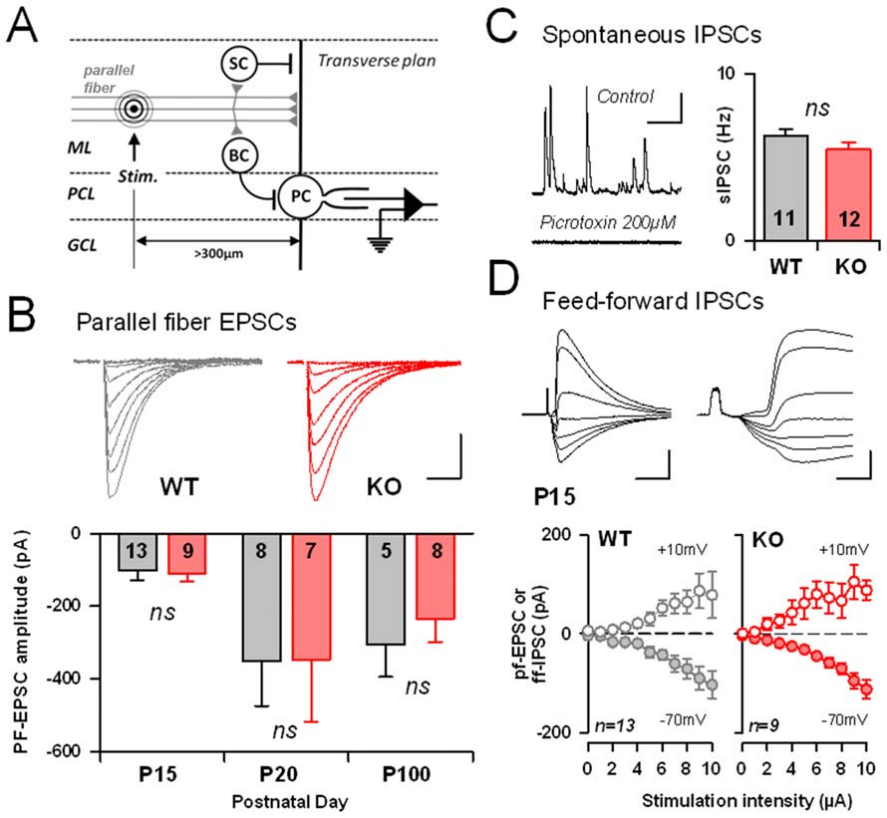
Cerebellum cortex electrophysiology in the absence or presence of TRAF4. (A): Scheme of the experimental procedure. Parallel fibers were stimulated by an electrode placed in the middle of the molecular layer (ML, Stim.) at more than 300 µm from the recorded Purkinje cell (PC). GCL: granule cell layer; PCL: Purkinje cell layer; SC: stellate cell; BC: Basket cell. (B): PF evoked EPSC at different stimulation strength were obtained from wild-type and TRAF4 KO mice, at different postnatal ages. No difference in the mean eEPSC at 10 µA was detectable. The number of recorded cells is indicated. NS: not significant. Scale bars: 20 msec and 200 pA. (C): The frequency of spontaneous inhibitory postsynaptic currents was similar between genotypes. sIPSCs were abolished by the GABAA receptor antagonist Picrotoxin (200 µM). Number of recorded cells is indicated. Scale bars: 0.5 sec and 100 pA. (D): At P15, both parallel fiber EPSC, and associated feed-forward IPSCs (ff-IPSC) had similar amplitudes between WT and KO mice at all stimulations tested. Ff-IPSCs and PF-EPSCs are recorded by holding the PC at +10 mV and −70 mV respectively. Scale bars: Left: 15 msec and 200 pA; Right: 3 msec and 200 pA.

The ataxia observed in the TRAF4-KO mice may therefore be due to modifications in the cerebellum microcircuitry. Indeed, alterations in Purkinje cell survival have been associated with rearrangements of the synaptic inputs onto the surviving cells. For example, Aldolase-C expression, which has been suggested to be correlated with parasagital heterogeneity in the feed-forward circuits of the cerebellum cortex [47], is also crucial for the survival of Purkinje cells [48]. Thus, to directly address the synaptic connectivity in the cerebellum cortex in the absence of TRAF4, we performed whole cell patch-clamp recordings of the Purkinje cells in acute transverse cerebellar slices of TRAF4-KO and WT brains [30] (Figure 4). During the early postnatal period, cerebellar granular cells migrate from the external granular cell layer to the internal cell layer to establish synaptic contacts with the Purkinje cells (Parallel fiber→Purkinje cell synapses). Thus, the loss of numerous Purkinje cells in the absence of TRAF4 may perturb the excitatory drive of parallel fibers onto the Purkinje cells [49]. However, this was not the case. The excitatory post-synaptic currents elicited by local stimulation within the molecular layer were similar in TRAF4-KO or WT Purkinje cells (Figure 4A and B).

The regularity of the spiking activity of Purkinje cells has been correlated with motor coordination and crucially depends on the activity of molecular layer interneurons (MLIs) [50]. Thus, we recorded the spontaneous (sIPSCs, Figure 4C) inhibitory transmission in Purkinje cells by holding Purkinje cells at +10 mV. Both the mean frequency (Figure 4C) and amplitude (data not shown) of sIPSCs were similar in 15-day-old (P15) TRAF4-KO and WT Purkinje cells. Further, when evoked by incoming parallel fiber excitation, feed-forward evoked IPSCs were also comparable between genotypes ([51], Figure 4D), indicating that both the synaptic weight at the MLI→PC synapses and the efficacy of the parallel fibers to recruit MLIs are preserved in the absence of TRAF4. Together, these electrophysiological results suggest that no major rearrangement of the cortical micro-circuitry in the remaining Purkinje cells occurs in the absence of TRAF4.

### TRAF4 deficiency favors the expression of the neurite outgrowth inhibitors Nogo A and MAG

**Figure 5:**
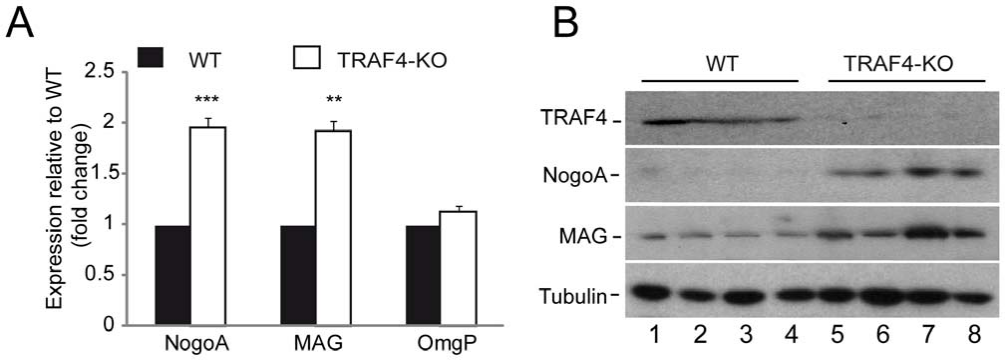
NogoA, MAG and OmgP Expression in the cerebellum of 8-week-old WT and TRAF4-KO mice. (A): Q-RT-PCR analysis of NogoA, MAG and OMgP expression in cerebellum total RNA extracts from WT (black) and TRAF4-KO (white) mice. Both NogoA and MAG were overexpressed whereas OmgP level remained stable. Data are means ± SD (n = 8) (ANOVA, Fischer's test). (B): Western blot analysis of TRAF4, NogoA, MAG and tubulin expression using 4 total protein extracts from WT (lanes 1–4) and TRAF4-KO (lanes 5–8) cerebellum. NogoA and MAG proteins were overexpressed in the absence of TRAF4. Tubulin served as loading control.

To further understand how TRAF4 deficiency could induce Purkinje cell death, we analyzed myelin-related proteins expressed by oligodendrocytes and known to induce neuron apoptosis. Myelin-associated glycoprotein (MAG), oligodendrocyte myelin glycoprotein (OmgP) and reticulon-4 (Nogo A) have been reported to be overexpressed following myelin alterations which in turn inhibit axon outgrowth, regeneration and/or repair [52]. We performed Q-RT-PCR analysis of Nogo A, MAG and OmgP mRNA expression in the cerebellum of TRAF4-KO and WT mice (6 animals/group) (Figure 5A). Nogo A and MAG levels were significantly increased in TRAF4-KO mice compared with WT (2.0±0.1 fold change, p<0.0001 and 1.9±0.2 fold change, p = 0.0062, respectively). OmgP expression was unchanged (1.1±0.1 fold change, p = 0.3624). Thus, the overexpression of NogoA and MAG in the absence of TRAF4 results from their transcriptional upregulation. NogoA and MAG overexpression was confirmed at the protein level by western blot analysis of total cerebellum protein extracts from WT (lanes 1–4) or TRAF4-KO (lanes 5–8) mice (Figure 5B). Altogether, these results indicate that TRAF4 deficiency leads to the activation of the neurite outgrowth inhibitory pathway.

### TRAF4 deficiency activates the NgR/p75NTR/RhoA signaling pathway

**Figure 6:**
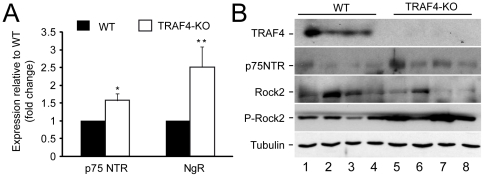
Activation of the NgR/p75NTR/RhoA signaling pathway in 8-week-old TRAF4-KO mouse cerebellum. (A): Q-RT-PCR analysis of p75NTR and NgR expression in WT (black) and TRAF4-KO (white) cerebellum total RNA extracts. Both receptor expression was increased in the absence of TRAF4. Data are means ± SD (n = 8) (ANOVA, Fischer's test). (B): Western blot analysis of TRAF4 and p75NTR and downstream effector Rock2 using cerebellum protein extracts from 4 WT (lanes 1–4) and 4 TRAF4-KO (lanes 5–8) mice. This confirmed the overexpression of p75NTR at the protein level, and its activity via RhoA since Rock2 exhibited higher phosphorylation levels (P-Rock2). Tubulin served as loading control.

In the CNS, there is a cross-talk between neurons and oligodendrocytes. It has been reported that NogoA and MAG proteins present at the oligodendrocyte membrane might interact with the Nogo receptor (NgR) located at the neuron membrane [53]. The NgR acts through binding to a co-receptor, the p75 neurotrophin receptor (p75NTR) [16]. Q-RT-PCR analyses of total RNA cerebellum extracts showed that NgR and p75 NTR mRNA levels were increased in TRAF4-KO mice compared with WT (2.5±0.6 fold change, p = 0.0019 and 1.6±0.2 fold change, p = 0.0189, respectively) (Figure 6A). Moreover, it has been reported in neuronal cells that p75NTR might activate the Ras homologous A (RhoA) signaling pathway to mediate cytoskeletal changes [16]. Accordingly, western blot analyses of cerebellum protein extracts showed that the phosphorylation level of the Rho-associated coiled-coil containing protein kinase 2 (Rock2), a key effector of RhoA, was increased in TRAF4-KO mouse cerebellum that expressed higher p75 NTR protein levels (lanes 5–8) compared with WT (lanes 1–4). Altogether, these data indicate an activation of the RhoA signaling pathway in the absence of TRAF4.

## Discussion

In the present study, we provide evidence that TRAF4 is a new player in the myelination processes, and a key factor in cerebellar ontogenesis/homeostasis. The locomotion/motor coordination deficits observed in TRAF4-KO mice are similar to that described for several spontaneous murine mutants exhibiting cerebellar ataxia [26].

We show that, in addition to neurons, TRAF4 is expressed by oligodendrocytes at all stages of their differentiation. Moreover, TRAF4 expression is an early event in oligodendrocyte differentiation since it is strongly expressed in early OPC, suggesting that TRAF4 might play a critical role in oligodendrocyte commitment.

Dramatic ultrastructural alterations of the TRAF4-KO CNS myelin were observed, including a lower number of myelinated axons, disorganized myelin layers and abnormal paranodes, indicating that TRAF4 is a key player in the CNS myelination processes. Myelin is essential for the effective and rapid propagation of action potentials and therefore, the functional integrity of the nervous system [39] [40]. Myelination is a multistep process. In the CNS, the myelin sheath is formed by the spiral wrapping of multiple layers of oligodendrocyte glia plasma membrane extensions around the axon, followed by the extrusion of cytoplasm and the compaction of the stacked membrane bilayers. The reciprocal communication between neurons and oligodendrocytes plays an essential role in the domain organization of myelinated axons, defining node, paranode, juxtanode and internode domains. The myelin phenotype observed in the absence of TRAF4 indicates that the septate-like junctions located between the myelinating glia and axonal membrane, and the TJ-like structures involved in the intermembranous sealing of glial cells, that both contribute to stabilization of correct compartmentalization, were altered. Consistently, the expression of caspr, a major transmembrane component of axoglial junctions in axons [38], and claudin-11, a member of the claudin family of TJ transmembrane proteins predominantly expressed by oligodendrocytes and that constitutes a marker for intramyelinic linear-array TJs in CNS [34], were reduced in TRAF4-KO cerebellum. Interestingly, it has been shown that the mice deficient for claudin-11 or caspr present myelin and TJ disorganization, alterations in locomotion coordination and/or severe ataxia [34] [35]. Our findings implicate the importance of TRAF4 in the formation and/or stability of axoglial and interglia junctions, and subsequent myelin integrity.

Depending on the organ and/or cell types, various transmembrane proteins are involved in the TJs to guarantee cell polarity: occludin is observed in epithelia, claudin-11 in oligodendrocytes, and caspr in neurons. The crucial task of TRAF4 in the polarity function of TJs is highlighted by the following data. First, TRAF4 was previously identified as a TJ-related protein in adjacent epithelial cells, responsible for the maintenance of the epithelium integrity [18]. Second, the present data yield new insights into the role of TRAF4 in the establishment and/or maintenance of axoglial and interglial TJs. Third, claudin-11 is also normally robustly expressed in Sertoli cells of the testis where it forms parallel organized TJs similar to those shown in oligodendrocytes [34]. Interestingly, the testis expresses TRAF4 [20], and exhibits altered TJs in TRAF4-KO mice (our unpublished results). Altogether, these data represent conclusive evidence for the pivotal contribution of TRAF4 to TJ structures and function, and suggest that TRAF4 shares a general function in several TJ types, presumably independent of the nature of the transmembrane proteins involved (e.g.: occludin, claudin, caspr). In this context, TRAF4 might act at the level of the cytoplasmic plaque, a scaffolding and signaling center, underlying TJs at the plasma membrane [54]. This macromolecular structure interacts with the cytoskeleton and recruits the integral membrane proteins that bind to adjacent cells. The present data further support the already proposed role for TRAF4 in cell polarity, tissue homeostasis, cytoskeletal organization and morphogenesis [14] [16].

Effects consecutive to myelin defects are mediated via the overexpression and release of myelin-associated inhibitory molecules into the axonal environment [52]. In this context, MAG and NogoA, which colocalizes with MAG in the innermost loop of the myelin membrane [55], are 2 prototypical myelin-derived neurite growth inhibitors. We show here that TRAF4 deficiency leads to increased MAG and NogoA expression. MAG and NogoA are glial ligands for the neuronal glycosyl-phosphatidylinositol (GPI)-anchored NgR that transduces the downstream growth inhibitory signal in susceptible neurons. This is achieved via NgR binding to the transmembrane co-receptor p75NTR, a member of the TNFR-superfamily that is expressed in neurons [53]. NgR/p75NTR has been shown to regulate cell morphogenesis by controlling the activity of RhoA, a member of the small GTPase Rho family. Moreover, activation of downstream signaling via p75NTR requires intracellular adaptor proteins since this receptor is devoid of intrinsic kinase activity. In this line, Rho guanine-nucleotide dissociation inhibitor (Rho-GDI) binds to the cytoplasmic domain of p75NTR to mediate the signal [16]. Finally, it has been reported that MAG or NogoA activate RhoA and its downstream effector Rho-associated kinase (ROCK) leading to actin cytoskeleton rearrangement favoring axon regeneration inhibition and neuron apoptosis [56] [57] [16]. In this context, the observed MAG, NogoA, NgR and p75NTR overexpression and up-regulated function, as evidenced by increased phosphorylation of Rock2, clearly indicate that TRAF4 deficiency activates the NgR/p75NTR/RhoA pathway. Collectively, these data strongly support that TRAF4 normally down-regulates this pathway. In this context, in addition to its role in TJs homeostasis, TRAF4 may also directly modulate p75NTR signaling pathway since it has been reported to bind with the p75NTR intracytoplasmic juxtamembrane region [12], [58].

Consistent with the activation of the RhoA pathway, we observed Purkinje cell apoptosis in TRAF4-KO cerebellum. The cerebellum is the brain region essential for coordination and motor control, and TRAF4-KO mice present motor behavior deterioration. In the process of motor coordination, Purkinje cells and more precisely their spontaneous discharge activity play an essential role [49]. The precision of the spiking pattern of Purkinje cells is controlled both by incoming cortical, vestibular and sensory information relayed by excitatory and inhibitory synaptic inputs [59], as well as intrinsic conductances [60]. For example, feed-forward recruitment of local interneurons has been shown to efficiently control Purkinje cell spiking activity [51] [30]. Although no major rearrangement of the cortical microcircuitry is induced by the absence of TRAF4 in the remaining TRAF4-KO Purkinje cells, the dramatic Purkinje cell degeneration observed in the TRAF4-KO cerebellum leads to the overall reduction in the number of functional units. Interestingly, several studies report that ataxia might be due to Purkinje cell number reduction [61] [48] [62]. Thus, the loss of Purkinje cells in TRAF4-KO mice may participate in the ataxic syndrome developed by these mice.

Finally, Rho GTPase signaling has been reported to substantially control polarity protein signaling and vice versa. The complex interplay between RhoA and polarity proteins is not fully understood [63]. It has been shown that junctional dynamics and RhoA activity are interconnected, at least in the epithelial cells. Indeed, on the one hand, activation of RhoA is implicated in the disassembly of TJs, most likely by regulation of the actin cytoskeleton. On the other hand, TJs might negatively regulate RhoA signaling as the TJ-associated Rho exchange factor (GEF-H1) contributes to the downregulation of active RhoA in confluent monolayers [64]. Thus, Rho activities need to be finely balanced to obtain optimal TJ integrity. Thus, our data bring new clues about the regulatory function of TJs on the RhoA signaling pathway, and identify TRAF4 as being a major molecule in this process.

We might hypothetize that TRAF4 should not act directly on the NogoA/MAG signaling pathway. Based on the essential function of TRAF4 on TJs and published data in the field of myelin alteration, we propose the following cascade of events to explain the TRAF4-KO ataxia phenotype : the absence of TRAF4 first affects myelin TJs leading to the loss of myelin integrity, and subsequently to MAG and NogoA overexpression, activation of the NgR/p75NTR/RhoA signaling pathway, actin cytoskeleton changes, Purkinje cell death and, hence, ataxia.

The present study will hopefully contribute to the understanding of demyelinating or neurodegenerative diseases. For example, genome-wide screens have identified several candidate mechanisms for schizophrenia, including myelination disturbances [65], and if future studies are needed to elucidate the molecular mechanisms that underlie these abnormalities, there is converging evidence for the implication of NgR [66]. Interestingly, a decrease of TRAF4 expression has been observed in the cortex from schizophrenic patients [67].
